# Feral Swine Commercial Slaughter and Condemnation at Federally Inspected Slaughter Establishments in the United States 2017–2019

**DOI:** 10.3389/fvets.2021.690346

**Published:** 2021-09-03

**Authors:** Judy Akkina, Howard Burkom, Leah Estberg, Lydia Carpenter, Morgan Hennessey, Karen Meidenbauer

**Affiliations:** ^1^United States Department of Agriculture, Animal and Plant Health Inspection Service, Veterinary Services, Fort Collins, CO, United States; ^2^The Johns Hopkins University Applied Physics Laboratory, Laurel, MD, United States; ^3^United States Department of Agriculture, Animal and Plant Health Inspection Service, Veterinary Services, Des Plaines, IL, United States; ^4^United States Department of Agriculture, Animal and Plant Health Inspection Service, Veterinary Services, Cheyenne, WY, United States

**Keywords:** feral swine, swine condemnations, slaughter inspection, slaughter monitoring, surveillance, condemnation reasons

## Abstract

Feral swine populations in the United States (US) are capable of carrying diseases that threaten the health of the domestic swine industry. Performing routine, near-real time monitoring for an unusual rise in feral swine slaughter condemnation will increase situational awareness and early detection of potential animal health issues, trends, and emerging diseases. In preparation to add feral swine to APHIS weekly monitoring, a descriptive analysis of feral swine slaughter and condemnations was conducted to understand the extent of commercial feral swine slaughter in the US at federally inspected slaughter establishments and to determine which condemnation reasons should be included. There were 17 establishments that slaughtered 242,198 feral swine across seven states from 2017 to 2019. For all 17 establishments combined, feral swine accounted for 63% of slaughtered animals. A total of 23 types of condemnation reasons were noted: Abscess/Pyemia, Arthritis, Contamination, Deads, Emaciation, General Miscellaneous, Icterus, Injuries, Metritis, Miscellaneous Degenerative & Dropsical Condition, Miscellaneous Inflammatory Diseases, Miscellaneous Parasitic Conditions, Moribund, Nephritis/Pyelitis, Non-ambulatory, Pericarditis, Pneumonia, Residue, Sarcoma, Septicemia, Sexual Odor, Toxemia, and Uremia. Exploratory analysis was conducted to determine which condemnation reasons should be included for weekly monitoring. For most condemn reasons, weeks of unusually high condemnations were noted. For example, a period of high pneumonia condemnations occurred from December 2, 2018 through February 3, 2019 with a spike on January 6, 2019 and a spike in dead swine occurred on November 3, 2019. The seasonal impacts on limited quality food resources, seasonal variation in the pathogen(s) causing pneumonia, and harsher weather are suspected to have an impact on the higher condemnation rates of pneumonia and dead swine during the winter months. Based on condemnation frequencies and the likelihood of enabling situational awareness and early detection of feral swine health emerging diseases, the following were selected for weekly monitoring: abscess/pyemia, contamination/peritonitis, deads, emaciation, injuries, miscellaneous parasitic conditions, moribund, pneumonia and septicemia. Detection of notable increases in condemnation reasons strongly suggestive of foreign animal or emerging diseases should contribute valuable evidence toward the overall disease discovery process when the anomalies are both confirmed with follow up investigation and combined with other types of surveillance.

## Introduction

**Figure 1:**
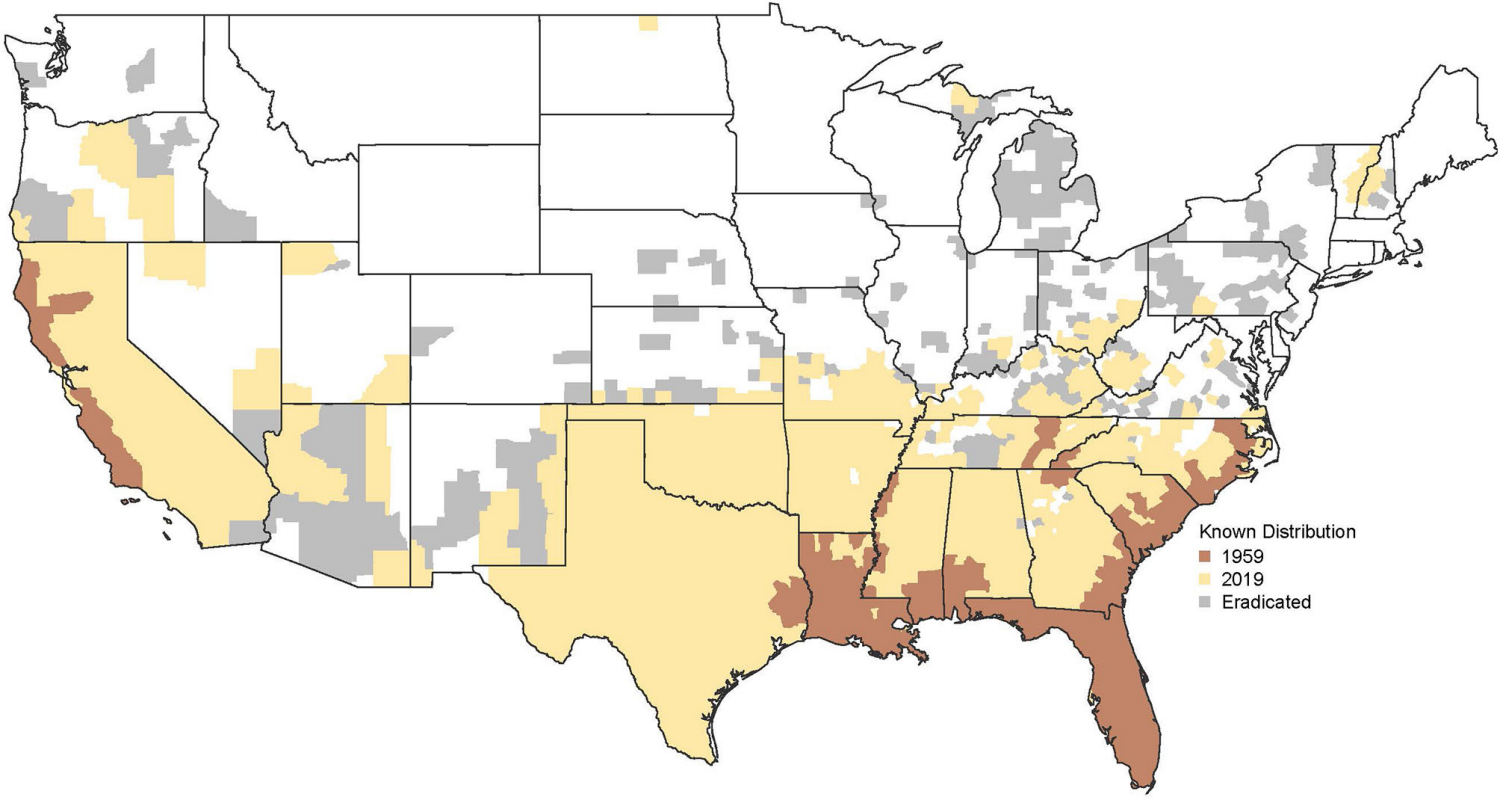
Change in county level distribution of feral swine in the contiguous United States from 1959 (brown) to 2019 (yellow). Gray indicates counties where pigs have previously occurred but are currently not present as a result of invasive species control activities. Data describing nationwide distribution (presence/absence) of feral swine at the county scale are from these sources: USDA (4), Corn and Jordan (5), Hanson and Karstad (6), and Waithman et al. (7). Data were processed using methods described in Miller et al. (8).

Feral swine are swine of a domesticated species that now live without direct human supervision or control (1). Over the last 60 years feral swine have become an increasing potential disease risk for domestic swine across the US (2). In 2016 there were an estimated 6.9 million feral swine in 36 US states (3). Figure 1 shows the change in the geographic extent of the feral swine population between 1959 and 2019. The map shows uniform distribution across California, southeastern and south-central US, outlier populations in several northern states, and successful eradication in some counties and states.

Feral swine cause destruction to natural vegetation and agricultural lands and are in contact with other wildlife, livestock, and their habitats. Globally these animals are known reservoirs of viral, bacterial and parasitic diseases that infect humans and other animal species. Feral swine can carry and propagate viral pathogens of significant concern including classical swine fever (CSF), foot-and-mouth disease virus (FMD), pseudorabies virus (PRV), African swine fever virus (ASFV), porcine circovirus type 2, porcine reproductive and respiratory syndrome virus (PRRS), porcine epidemic diarrhea virus (PEDv) and porcine parvovirus. Common feral swine bacterial disease threats include brucellosis, salmonellosis and Q-fever, and parasitic disease threats include *Trichinella* sp., *Toxoplasma gondii* and *Taenia solium* (2, 9). Efforts to control feral swine populations vary from state to state and include regulations on transport and release, hunting and bounty programs, and commercial slaughter. Swine trapping methods using pens are generally similar across the states (10). The definitions, or categories for feral swine have ranged between game, wild, nuisance, outlaw, and exotic animals (10). As wild animals, feral swine are likely to suffer stress, potential pain, and fear during trapping and transport. Additionally, trappers who consider feral swine nuisance or outlaw animals may add to that fear and stress through their handling practices. Some states such as Kansas and Ohio preclude hunting and commercial slaughter because these activities can provide an incentive to transport, maintain or increase populations (11, 12). Nationally, the United States Department of Agriculture (USDA) Animal and Plant Health Inspection Service (APHIS) National Feral Swine Damage Management Program seeks to protect agricultural and natural resources, property, animal health, and human health and safety by managing damage caused by feral swine in the US and its territories (13). One objective of this program is to monitor feral swine for pathogens that affect domestic swine, other livestock and human health.

Consumer demand for wild game meat, including wild boar, creates an emerging market and economic opportunity for commercial sale (14). The USDA Food Safety and Inspection Service (FSIS) considers feral swine of the species *Sus scrofa* as an amenable swine subclass (15), and therefore subject to federal or state inspection requirements according to the Federal Meat Inspection Act (16). If meat from feral swine is to be sold, whether to individuals, restaurants, or retail stores, it must undergo federal or state inspection. Only meat under federal inspection can enter interstate commerce or be exported, with an exception for nine states (Iowa, Indiana, Maine, Missouri, North Dakota, Ohio, Vermont, Wisconsin, and South Dakota) that participate in the Cooperative Interstate Shipment program (17, 18). However, with the exception of Missouri and Vermont, feral swine populations in these states are negligible. Inspection requirements include ante- and post-mortem inspection. Ante-mortem inspection requires that animals are brought to the slaughterhouse alive, therefore meat products from feral swine killed in the field cannot enter commerce.

To enable and facilitate commercial feral swine slaughter, state regulations are required to permit the capture and transport of the animals to a slaughterhouse. As the state with the largest feral swine population, Texas has developed an innovative system to address its feral swine issue. The Texas Animal Health Commission (TAHC) is responsible for regulating the movement of feral swine, feral swine holding facilities, and some aspects of hunting preserves (19). Once trapped swine may be held for up to 7 days in an escape-proof pen or trailer (20). Then the swine can legally be moved only to a recognized slaughter facility, authorized hunting preserve, or an approved holding facility (21). Neither Texas or Florida indicate requirements for protection from the elements on their feral swine holding facility applications (22, 23) and depending on the holding pen arrangements, animals may be housed at a high stocking density. Florida and Oklahoma have also adopted the Texas feral swine model for trapping, holding facilities, and movement restriction (24, 25).

Since 2011, the USDA, APHIS, Veterinary Services (VS) has conducted weekly monitoring of USDA, FSIS data on slaughter and condemnations by swine subclass, including market (typically 220–260 lbs.), roaster (<220 lbs., for whole carcass roasting), sows and boars (typically over 400 lbs.). Tracking of feral swine slaughtered at federally inspected slaughter establishments became possible after FSIS implemented Notice 78-16 to designate feral swine as a new subclass in the FSIS Public Health Information System (PHIS) on October 16, 2016 (26). With the increasing global spread of ASFV, the continuing threat of CSF and FMD and the monumental economic impacts these diseases could have on the US swine industry, up-to-date monitoring of the feral swine population has become very important for detecting the entry of ASFV into the US to enable prompt outbreak mitigation, response and control. The unrestricted movement of feral swine between states allows them to spread diseases that they may acquire, as has been seen with the ASFV outbreaks in Europe. Given the complexities in feral swine management, movement, and slaughter, monitoring of this high-risk population is an essential component of US preparedness for highly transmissible diseases of swine. An approach that can be used to perform routine near-real time monitoring for an unusual increase in feral swine slaughter condemnation will increase situational awareness and early detection of potential animal health issues, trends, and emerging diseases. An improved understanding of commercial slaughter and condemnation data is needed to allow for the development of monitoring targets and action thresholds. In preparation to add feral swine to APHIS weekly monitoring, a descriptive analysis of feral swine slaughter and condemnations was conducted to understand the extent of commercial feral swine slaughter in the US at federally inspected slaughter establishments and to determine which condemnation reasons should be included in monitoring.

## Materials and Methods

FSIS is responsible for inspecting swine to be slaughtered for meat that will enter interstate and foreign commerce (27, 28). Inspection at a slaughter establishment begins in the ante-mortem area where FSIS inspection program personnel (IPP) inspect all live animals to determine whether they are fit for human consumption using standard inspection procedures (29). IPP must also verify that all livestock that are brought onto official establishment premises are humanely handled (28). In most cases, IPP are to perform ante-mortem inspection once livestock are offered for slaughter using the steps in the FSIS directive (29). However, inspection personnel may perform ante-mortem inspection on livestock that have not been placed in pens (e.g., alleyways) or unloaded from transport vehicles (e.g., injured livestock). It is also a requirement for slaughter establishments to notify IPP when they want animals inspected prior to slaughter throughout the day. Animals that arrive dead or die in the pens are tagged as “U.S. Condemned.” Animals showing signs of illness may be identified as “U.S. Suspect” and segregated until the animal has received additional inspection by an FSIS veterinary medical officer (VMO) (28).

The purpose of post-mortem inspection is to protect the public's health by ensuring that the carcasses and parts that enter commerce are wholesome, not adulterated, and properly labeled. IPP must perform a careful post-mortem examination and inspection of the carcasses and parts of each animal using standard inspection procedures (30). Inspectors look for signs of disease or pathological conditions that would render a carcass or part unwholesome or otherwise unfit for human consumption. Any carcass in need of disposition is segregated until a final inspection can be completed (31).

The FSIS Public Health Information System (PHIS) contains swine slaughter inspection information entered by IPP, including the counts of swine slaughtered and condemned along with the reason for condemnation (29, 30). The reasons for both ante- and post-mortem condemnation are standardized and selected from a list. The USDA, APHIS, VS has a Memorandum of Understanding with FSIS to allow access to slaughter and condemnation data to monitor emerging animal health issues (32).

Feral swine slaughter and condemnation data aggregated by week for 2017–2019 were downloaded from the PHIS as a text file. Data fields included establishment ID, weekly counts of swine slaughtered by swine subclass, weekly condemnation counts by swine subclass, condemnation reason, and ante or post-mortem condemnation. Microsoft Access 2013 was used for data storage, and Rstudio 1.2.1335 with R x64 3.6.0 and Microsoft Excel Professional Plus 2016 for descriptive analyses.

During 2017–2019 (the dates between 1/1/2017 and 1/4/2020 were included because the data aggregated by week were downloaded) there were 17 FSIS inspected slaughter establishments in seven states that slaughtered feral swine for at least 1 week. Over the 3 years (2017–2019), 242,198 feral swine were slaughtered at these 18 establishments, located primarily in the southeastern US where much of the feral swine population is located. Establishments were in the following states: six in Texas, four in Florida, three in Georgia, two in Hawaii, and one each in Kentucky, and South Carolina. One establishment in Georgia only slaughtered feral swine 1 week during 2017–2019.

Exploratory analysis was conducted to determine which condemnation reasons should be included, along with aggregating counts appropriately, to focus on principal condemnation reasons and trends for weekly monitoring. The condemnation totals were 1,590 post-mortem condemns and 375 ante-mortem condemns during 2017–2019 (the dates between 1/1/2017 and 1/4/2020 were included because the data aggregated by week were downloaded). A total of 23 types of condemnation reasons were noted: Abscess/Pyemia, Arthritis, Contamination, Deads, Emaciation, General Miscellaneous, Icterus, Injuries, Metritis, Miscellaneous Degen. & Dropsical Condition, Miscellaneous Inflammatory Diseases, Miscellaneous Parasitic Conditions, Moribund, Nephritis/Pyelitis, Non-ambulatory, Pericarditis, Pneumonia, Residue, Sarcoma, Septicemia, Sexual Odor, Toxemia, and Uremia. Explicit condemnation categories and criteria are documented for each of these reasons in the appropriate US Code of Federal Regulations.

The t-test to assess the equality of condemnation to slaughter proportions across years and locations was performed. Additionally, a regression trend line and a two-sided Kolmogorov-Smirnov (K-S) hypothesis test were used to assess the seasonality in both monthly slaughter and condemnation counts.

## Results

In preparation to add feral swine to APHIS weekly monitoring processes, a descriptive analysis of feral swine slaughter and condemnations was conducted to understand the extent of commercial feral swine slaughter in the US at federally inspected slaughter establishments. Table 1 represents slaughter counts of feral swine and all subclasses combined (market, roaster, feral, sow and boar) for these 17 establishments, with feral swine accounting for 63% of their slaughtered animals overall. However, the percentage of feral swine slaughtered varied widely by establishment. The proportion of feral swine slaughtered at the Texas establishments inspected by FSIS was much higher (80%) than in the other states (2–4%). Condemn counts and percentages by year are represented in Table 2. At the Texas establishments, the percentage of feral swine condemned was consistent over the 3 years at 0.8%. The percentage condemned at establishments in other States was more variable, ranging from 0 to 1.7%. In the other States, the percentage condemned between the years 2017 vs 2018 and 2018 vs 2019 were significantly different, while not so for 2017 vs 2019 (t-test alpha = 0.01).

**Table 1:** Counts of all swine slaughtered and proportion of feral swine at 17 FSIS inspected slaughter establishments that slaughtered feral swine, 2017–2019.

	**Establishment state location (# of plants)**				
	**Florida (4)**	**Georgia (3)**	**Texas (6)**	**All other states (4)**	**Total**
Count of all swine slaughtered	54,449	2,796	299,621	25,639	382,505
Count of feral swine slaughtered	2,177	92	239,338	591	242,198
Percent of slaughters that are feral swine	4%	3%	80%	2%	63%

**Table 2:** Feral swine total condemn and slaughter counts.

	**Year and state**					
	**2017**		**2018**		**2019**	
	**Texas**	**Other**	**Texas**	**Other**	**Texas**	**Other**
Condemn count	698	24	650	0	589	4
Slaughter count	85,609	1,414	81,808	1,105	71,921	341
Condemn percentage	0.82%	1.70%	0.79%	0.00%	0.82%	1.17%

**Figure 2:**
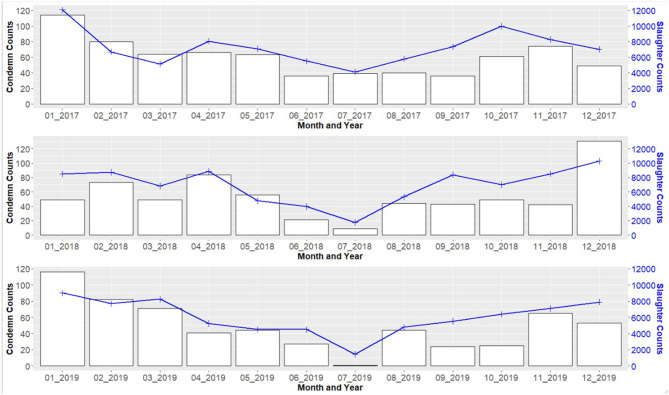
Feral swine slaughter and condemnation counts by month, 2017–2019.

**Figure 3:**
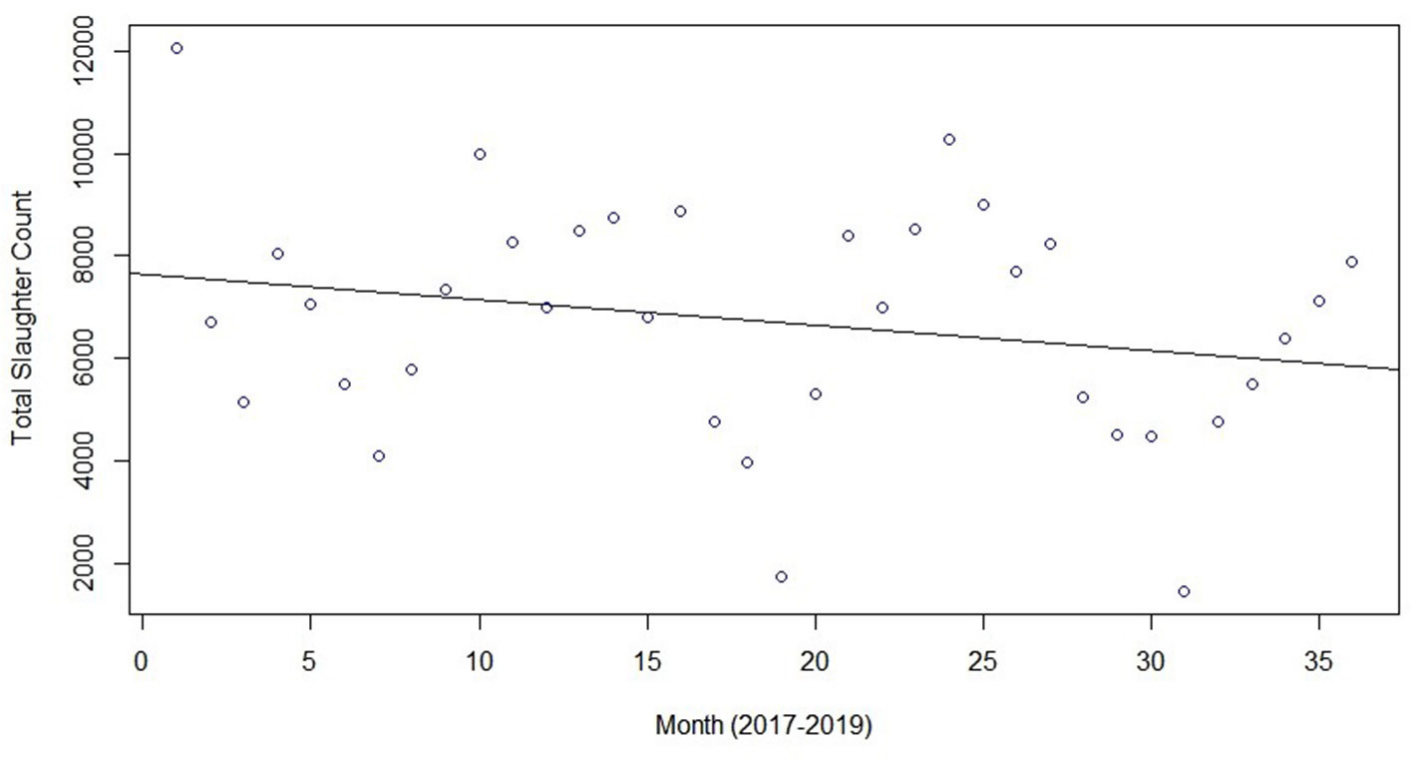
Time series of monthly total slaughter counts with trend line, 2017–2019.

**Figure 4:**
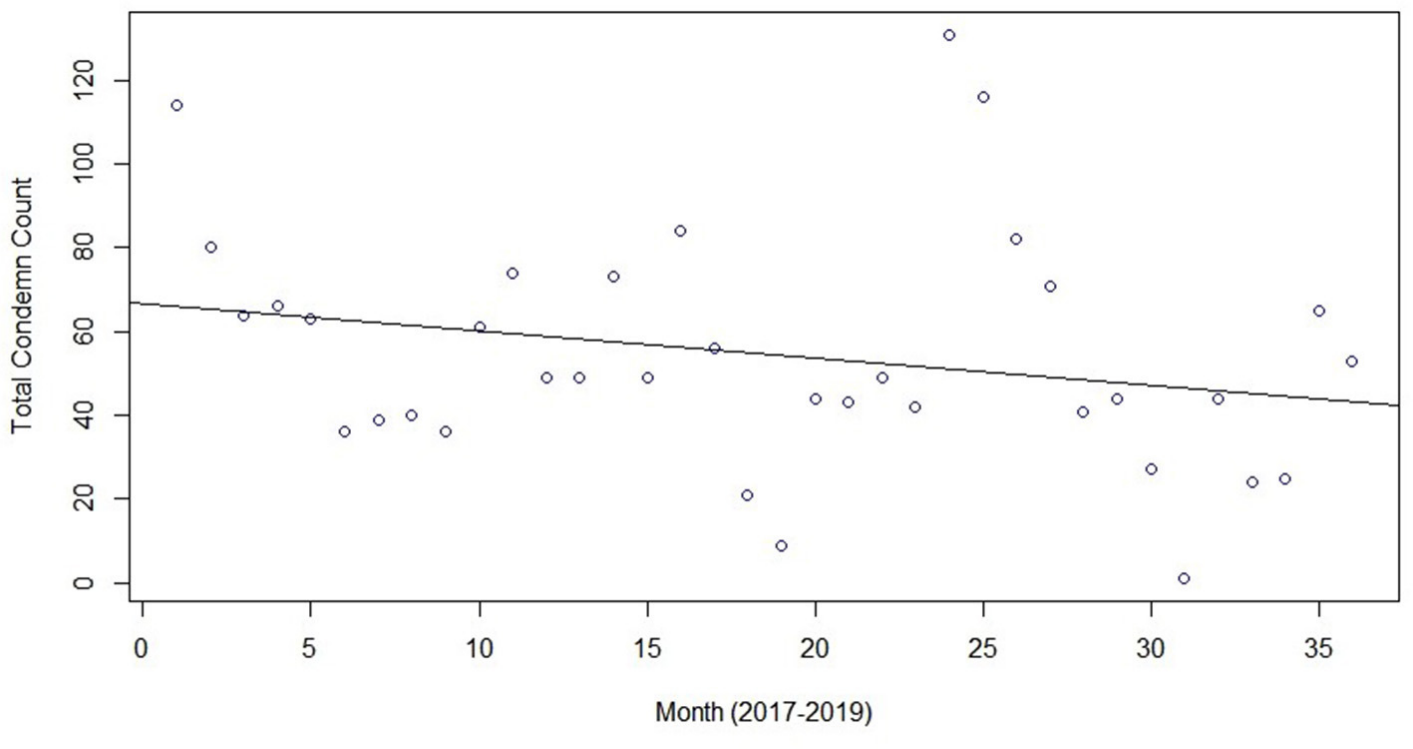
Time series of monthly total condemnation counts with trend line, 2017–2019.

Slaughter and condemn counts by month are shown in Figure 2, with one plot for each data year. Slaughter and condemnation counts tended to be highest in the early winter months of December and January and lowest in the early summer months of June and July. As a test of seasonality, we applied the two-sided Kolmogorov-Smirnov (K-S) hypothesis test to the 3-year time series of both slaughter and condemn counts. The null hypothesis was that the time series could be explained as a simple 3-year trend. Figures 3 and 4 present plots of the two time series with trend lines. We applied the K-S test comparing the respective pairs of time series and trend line and, for monthly data aggregation, considered an alpha threshold of p=0.05 as sufficient to reject the simple trend hypothesis. The K-S p-values were 0.008096 for the slaughter count time series vs trend and 0.00864 for the condemn count time series vs trend, thus rejecting the simple trend hypothesis while supporting the seasonality impression. More seasons of data from multiple test regions would permit more conclusive seasonality testing. Across all states, in the peak slaughter months, condemns were 1% of slaughter, while the percentage dropped to 0.6% in June and the lowest (0.5%), was in September.

An additional part of this study was to determine which condemnation reasons should be included in monitoring. Ante-mortem and post-mortem counts of condemnations are tabulated by reason in Table 3. Rows of the table are restricted to condemnation reasons listed more than ten times in the 3 years of data. The most frequent reasons for condemnation were pneumonia and abscess/pyemia. Aggregating counts appropriately was included in this exploratory phase of the study. Definitions and descriptions of the criteria for the condemnation reasons included in Table 3 are summarized below:

**Table 3:** Total counts of 10 most frequent reasons for feral swine slaughter condemnation, 2017–2019.

**Condemnation reason**	**Ante-mortem count**	**Post-mortem count**	**Total condemn count**
Pneumonia	0	400	400
Abscess/Pyemia	19	312	331
Contamination*	0	321	321
Deads	273	0	273
Injuries	3	256	259
Peritonitis	0	174	174
Moribund	77	0	77
Misc. parasitic conditions	3	48	51
Emaciation	0	24	24
Septicemia	0	21	21

* *Fecal material, urine, bile, hair, dirt or foreign matter*.

Pneumonia: Condemnation due to pneumonia may occur when there is acute inflammation of the lungs and pleura and the carcass is so infected that the consumption of the products may cause food poisoning (33). Pneumonia is an inflammatory condition of the lungs that may be caused by infectious agents, parasites, physical trauma, or foreign material inhalation. Ante-mortem findings may include discharge from nostrils (serous to mucopurulent discharge), cachexia, and pulmonary distress. Ante-mortem condemnation should occur if there are signs of marked pulmonary distress, or there is a combination of pneumonia respiratory signs and cachexia or moribund. Post-mortem findings may include hyperemia—increased blood flow in pulmonary vessels; red hepatization—lung is heavy, firm, and “liver-like” due to hyperemia, hemorrhage, edema, and leukocytes; gray hepatization—fibroplasia into areas of red hepatization; consolidation—chronic areas where fibroplasia is being organized; and lymph nodes draining lungs may be swollen and hemorrhagic.

Abscesses: Any organ or other part of a carcass that is affected by an abscess must be condemned. When the lesions are to such an extent as to affect the entire carcass, the entire carcass must be condemned (34). Abscesses are localized, “walled off” areas of pus. Ante-mortem findings may include swellings evident in various parts of the animal. Ante-mortem condemnation should occur if any combination of significant findings is present that would give evidence that the carcass would be condemned on post-mortem [e.g., abscesses as well as generalized (systemic) signs]. Post-mortem findings may include abscesses in various parts of the carcass or organs and localized, acute or chronic, reactive, or edematous lymphadenitis. Although pyemia may have caused them, multiple, localized, encapsulated abscesses about the body should not be confused with an active pyemia.

Pyemia: Condemnation due to pyemia may occur when pyemia, whether puerperal, traumatic, or without evident etiology, is present and the carcass to be so infected that the consumption of the products may cause to food poisoning (33). Pyemia is a variant of septicemia caused by pus-forming bacteria in which secondary foci of suppuration occur and multiple abscesses are formed. Fever, chills, sweating, jaundice and abscesses in various parts of the body mark the condition. Ante-mortem findings may include depression, lethargy, and swollen joints. Ante-mortem condemnation should occur if any combination of significant findings that would give evidence that the carcass would be condemned on post-mortem. Post-mortem findings may include acute suppuration (developing foci of suppuration) occurring as a result of pyogenic organism's entry into the systemic circulation, infarcts accompanied by acute suppuration, and generalized, acute, reactive, and edematous lymphadenitis.

Contamination: During the processing steps, carcasses, and organs must be handled in a sanitary manner to prevent contamination with fecal material, urine, bile, hair, dirt, or foreign matter. If contamination occurs, it must be immediately removed. Official swine slaughter establishments must also maintain written procedures to prevent the contamination of carcasses by visible fecal material, ingesta, and milk during slaughter and dressing processes (35).

Peritonitis: Condemnation due to peritonitis may occur when acute inflammation of the peritoneum is present and the carcass to be so infected that the consumption of the products may cause food poisoning (33). Peritonitis is a condition marked by inflammatory processes affecting the peritoneal lining which is usually caused by infectious agents. However, it can be initiated by ruptured bladder or other irritants. Post-mortem findings may include pathologic hemorrhage, generalized, acute lymphadenitis, degeneration of tissues or organs, and accumulation of fluid in abdominal cavity.

Deads: Livestock that are found to be dead or dying at an official slaughter establishment are identified as U.S. Condemned (36).

Moribund: Moribund animals are at the point of death, or those in a comatose or semicomatose condition, are identified as U.S. Condemned (36).

Ante-mortem injuries: Ante-mortem injuries may cause bruising and musculoskeletal damage. Any organ or part of a carcass that is badly bruised must be condemned. When the lesions are to such an extent as to affect the entire carcass, the entire carcass must be condemned (34). Injury ante-mortem findings may include impaired function such as non-ambulatory disabled or a lame animal, fractures, dislocations, abrasions, wounds, and hematomas. Ante-mortem condemnation should occur if injured animals show signs of generalized (systemic) effects. Post-mortem findings may include septic inflammations, localized recent bruises, injury, or fracture with hemorrhage into the tissues.

Miscellaneous Parasitic Conditions: Carcasses with excessive swine tapeworm (Cysticercus cellulosae) infestation must be condemned (37). For parasites not transmissible to humans, if parasites are distributed throughout a carcass so that their removal and the removal of the lesions caused by them is impracticable, then no part of the carcass can be passed for human consumption. If the infestation is excessive, the entire carcass must be condemned (38):

Emaciation: Carcasses may be condemned due to emaciation when the carcass is too emaciated to produce wholesome meat. When serous infiltration of muscle tissues, or a serous or mucoid degeneration of the fatty tissue is present on a carcass, it must be condemned (39). Emaciation is a condition that develops because of a low intake of food or an increase in the metabolic rate that causes the animal to deplete its normal body fat and protein reservoir. As this depletion becomes more pronounced, a typical abnormal physiological change in the fat and muscle tissues occurs. Emaciation is purely a post-mortem descriptive term and does not in any way apply to ante-mortem inspection. Post-mortem findings may include serous infiltration and degenerative change of muscular tissue and/or virtually all visceral and body fat.

Septicemia: Septicemia may be present as a complication of local necrosis (40). Condemnation due to septicemia occurs when the carcass to be so infected that the consumption of the products may cause food poisoning (33). Septicemia is syndrome of septic bacteremia accompanied by fever, hemorrhage, and severe systemic illness associated with the presence and persistence of pathogenic microorganisms or their toxin in the blood. It is frequently associated with some focus of inflammation that provides a continuing supply of organisms. Ante-mortem findings may include hyperemia of skin and dyspnea. Ante-mortem condemnation should occur when it is possible to establish a diagnosis of septicemia based on any combination of significant findings that would give evidence that the carcass would be condemned on post-mortem. Post-mortem findings may include infected wounds or bruises, generalized, acute lymphadenitis, degeneration of tissues or organs, and acute infarction.

Out of 375 ante-mortem condemnations, 350 were classified as Dead or Moribund with no other information available, so we chose to combine these reasons for condemnation into a “Deads/Moribund” category for weekly monitoring. Team discussion of the preliminary findings also led to combining the peritonitis and contamination (with fecal material, urine, bile, hair, dirt or foreign matter) condemnations to a single time series for weekly monitoring, since peritonitis, and associated gastrointestinal mucosa weakening and rupture, can lead to contamination. Based on condemnation frequencies and the likelihood of each enabling situational awareness and early detection of feral swine health emerging diseases, discussion of these analyses led to selection of the following reasons for weekly monitoring: abscess/pyemia, contamination/peritonitis, deads/moribund, emaciation, injuries, miscellaneous parasitic conditions, pneumonia and septicemia.

**Figure 5:**
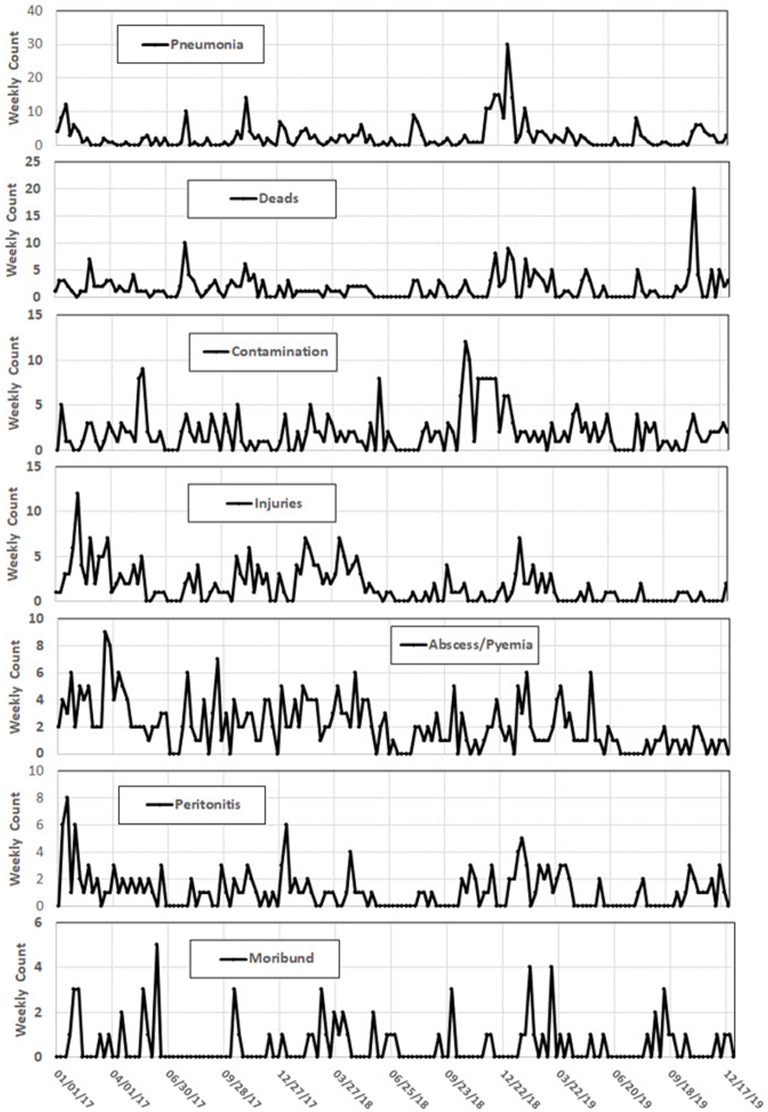
Weekly count plots of common reasons for feral swine slaughter condemnation, 2017–2019.

Figure 5 represents plots of the weekly counts from January 2017 through December 2019 for the 7 most frequent reasons in Table 3. For most condemn reasons, weeks of unusually high condemnations were noted. For example, a period of high pneumonia condemnations occurred from December 2, 2018 through February 3, 2019 with a spike on January 6, 2019 (Figure 5) and a spike in dead swine occurred on November 3, 2019 (Figure 5).

## Discussion

Feral swine were originally brought to the US for food and sport hunting and over the last several decades their population has been rapidly increasing and their geographic range across the US has continued to expand. Currently in some states, feral swine collected alive may be taken to a federally inspected slaughter establishment and processed as meat for human consumption. Compared to domestic pigs, wild boars have darker, less tender, and leaner meat (41). This study provides the first data on the extent of feral swine slaughter at federally inspected establishments in the US. Most of this slaughter (99%) occurred at six establishments in Texas. This is not surprising since Texas has the largest feral swine population in the US and a well-developed system in place to enable trapping, holding and transporting of feral swine to slaughter establishments. The most frequent reasons for condemnation at slaughter were pneumonia, abscess/pyemia, contamination/peritonitis, deads, injuries, moribund, miscellaneous parasitic conditions, emaciation and septicemia; all selected for monitoring.

Feral swine populations are a reservoir for PRV in the US. In a 2018 study the positive serology for PRV in Florida feral swine was 51 and 7% of the animals were viral shedding (42). This study indicates that a large proportion of the population is at least exposed to the herpesvirus, and the disease may recrudesce when the animals are stressed. PRV has a respiratory presentation so it could be a contributing factor to the pneumonia condemnation. An additional factor to consider is the potential maturity of feral swine trapped given the fact that 80% of commercial swine older than 22 months show pneumonic lesions at slaughter (43). Neither Texas or Florida indicate requirements for protection from the elements on their feral swine holding facility applications (22, 23). Therefore, exposure to the elements could predispose the animals to developing pneumonia or arrive at the slaughter plant in a moribund state. Depending on the holding pen arrangements, animals may be housed at a high stocking density and exposed to high levels of air contaminants, which has been correlated with presence of pneumonia and pleuritis at slaughter (44).

Feral swine are not vaccinated, therefore susceptible to diseases that we protect confinement-raised swine from (e.g., Leptospirosis, Erysipelas). These diseases could create clinical signs that require condemnation. Additionally, feral swine are not dewormed and are exposed to parasites that confinement-raised swine are not (e.g., kidney worm exposure through access to earthworms). Swine raised in confinement systems also have minimal physical risks in their environment (flat, slatted floors, no natural obstacles), but feral swine may have been exposed to predators, traps, and natural injuries while wild. These injuries, in addition to ones sustained during the trapping/capture, housing, and transportation process could increase the frequency of condemnation due to injury. Additionally, capture myopathy may be a cause of moribund condemnation. Capture myopathy has been defined as “a non-infectious disease of wild and domestic animals in which muscle damage results from extreme exertion, struggle, or stress,” and described to “occur naturally when prey animals are attempting to avoid predation, which is usually caused by humans. This is because these animals are adapted to escape from predators but are not adapted to struggle for long periods of time in man-made restraints. Capture myopathy occurs when animals overexert themselves (struggling in a trap for example) so much that physiological imbalances develop and result in severe muscle damage.” (45).

To enable and facilitate commercial feral swine slaughter, state regulations are required to permit the capture and transport of the animals to a slaughterhouse. While swine trapping methods in a pen are generally similar across the states (10), there may be varying circumstances surrounding the trapping (20, 25). The Texas Administrative Code states, “Feral swine that have been trapped and are being held for transportation to an authorized location, as provided by this subsection, may be held in an escape-proof cage on the vehicle or trailer that transported them from their original premise, or held within the transport trailer itself for up to seven days.” (46). Housing feral swine for 7 days in a trailer would likely predispose them to heat stress, potentially overcrowding, limited food and water, etc. Pneumonia is a longer-term condition and would take up to 7 days to develop. Given the TAHC time limit for detaining feral swine after trapping (20), pneumonia could develop in the timeframe between trapping, penning at a holding facility, and movement to a slaughter facility. Similar trapping time limit regulations do not appear to apply in the state of Florida (25), so the variability of the conditions following trapping may have a greater impact on these feral swine. Neither Texas or Florida indicate requirements for protection from the elements or time limits on their feral swine holding facility applications (22, 23). The potentially variable, confounding factors of feral swine holding lengths of time and conditions should be expected when considering the usefulness of condemnation reasons such as pneumonia, injury, or moribund as early indicators of diseases of interest.

Slaughter counts of feral swine and all subclasses combined indicate that the Texas establishments focus primarily on processing feral swine, whereas for the establishments in other states, feral swine slaughter is secondary to slaughtering of other subclasses (Table 1). There are FSIS directives providing instructions for following standard inspection procedures, for example FSIS IPP are to perform ante-mortem inspection by observing all livestock both at rest and in motion (29, 30). Descriptions for determination and classification of the reason for condemnation of animals were extracted from the US Code of Federal Regulations, as listed above. This documentation minimizes the variation in inspection procedures and how condemnation reasons are classified across slaughter establishments and IPP. The approach used for addressing this potential confounding effect on monitoring is to contact the appropriate IPP with questions about scenarios surrounding monitoring alerts.

Animal stress during housing and handling at a slaughter establishment likely occurs and may vary among facilities. It is the establishment's responsibility to follow the Humane Methods of Slaughter Act (28). FSIS VMOs and other IPP are responsible for verifying that establishments are complying with the Act (47). Noncompliance records (NRs) for humane handling can be issued when the violation is either extreme or less than egregious, such as not having water available in ante-mortem pens. This potential source of stress and adverse animal handling may have a confounding effect on the incidence of injuries as a cause for condemnation across slaughter establishments.

Of note is that all emaciation condemnations occurred post-mortem. That is because emaciation is a post-mortem descriptive term (39). Additionally, relatively few ante-mortem condemnations occurred with injured animals. This is because seriously crippled animals and non-ambulatory disabled swine are identified as U.S. Suspects, not U.S. Condemned (48).

Slaughter and condemnation counts tended to be highest in the early winter months of December and January and lowest in the early summer months of June and July (Figure 2). Across all states, in the peak slaughter months, by volume of feral swine, condemns were 1% of slaughter, while the percentage dropped to 0.6% in June and the lowest (0.5%), was in September. A combination of high pneumonia and dead condemnations during the early winter months contributed to this trend. Among the reasons for the summer drop-off in slaughters is the traditional July closure for maintenance of one of the large feral swine slaughter establishments in Texas (M. Bodenchuk, personal communication, March 23, 2020). In addition, the summer heat leads to higher death loss during transport, and so summer trapping and transport are infrequent.

For most condemn reasons, weeks of high condemnations, compared to other weeks in the data set analyzed, are noted. For example, a period of high pneumonia condemnations occurred from December 2, 2018 through February 3, 2019 with a spike on January 6, 2019 (Figure 3) and a spike in dead swine occurred on November 3, 2019 (Figure 3). The environmental impacts of limited quality food resources, seasonal variation in the pathogen(s) causing pneumonia, and harsher weather are suspected to have an impact on the higher condemnation rates of pneumonia and dead swine during the winter months. Texas and Florida can get cold and wet in the winter and it does not appear that these animals are required to be protected from the elements while in holding facilities (22, 23). The animal handling practice during cold, wet months could predispose the animals to weather-related health consequences. Weather-related stress, combined with animal crowding in the holding facilities, could predispose them to opportunistic diseases, such as Mycoplasma pneumonia. A change of weather is included as a predisposing factor for Mycoplasma pneumonia in the Merck Veterinary Manual along with the fact that the effects of the disease is “enhanced when large numbers of pigs are closely confined in poorly ventilated buildings under poor husbandry conditions” (49), which is likely how feral swine are housed in holding facilities. Another potential reason for higher condemnation rates of pneumonia and dead swine during the winter months is that holding facilities, transportation companies, and slaughter establishments may be closed around the holidays (December and January) and animals may not get sent to slaughter as soon as they would under normal circumstances, allowing health conditions to develop or worsen, allowing health conditions to develop or worsen.

Using information from this study, APHIS staff began weekly monitoring of the most frequent condemnation reasons to enable situational awareness and early detection of feral swine health issues, trends and emerging diseases. For example, a relatively high Contamination condemnation frequency may serve as an early indicator of presence of an enteric disease, such as Porcine Epidemic Diarrhea (PED) or Transmissible Gastroenteritis (TGE). Gastrointestinal diseases can damage or weaken intestinal mucosa, predisposing it to rupture and causing contamination of the carcass during carcass processing. Relatively high Deads condemnation frequency may serve as an early indicator of ASFV, Anthrax, CSF, Porcine Reproductive and Respiratory Disease (PRRS), or PED. A relatively high Emaciation condemnation frequency may serve as an early indicator of ASFV, CSF, PED, PRRS, Swine Vesicular Disease (SVD), or Tuberculosis. A relatively high Moribund condemnation frequency may serve as an early indicator of ASFV, CSF, Influenza, PED, PRRS, or PRV. A relatively high Pneumonia condemnation frequency may serve as an early indicatory of ASFV, Influenza, PRV, PRRS, or Anthrax. A relatively high Septicemia condemnation frequency may serve as an early indicator of ASFV, CSF, PRRS, or Anthrax.

During weekly monitoring operation APHIS VS analysts follow up on unusual condemnation spikes with the FSIS VMO at the establishment to gather more information. In addition, APHIS VS swine staff contacts the field epidemiologist in the state where the establishment with an unusual condemnation spike is located and APHIS VS field operations (FiOps) veterinarians follow up through local channels to obtain information from the local FSIS VMO and/or establishment management to obtain explanations for the spike. On rare occasions the VMO or plant management may notify APHIS or the State Veterinarian when a high mortality event occurs and FiOps staff in consultation with swine staff decide whether to conduct sampling based on the epidemiology surrounding the event. Despite small condemnation rates (0.5–1.0%), it is believed that performing near-real time monitoring for unusual increase in these rates can raise situational awareness and early detection of potential animal health issues, trends, and emerging diseases. Detection of notable increases in condemnation reasons strongly suggestive of foreign animal or emerging diseases should contribute valuable evidence toward the overall disease discovery process when it is both confirmed with follow up investigation and combined with other types of surveillance.

## Data Availability Statement

The data analyzed in this study is subject to the following licenses/restrictions: the data analyzed may not be released to the public. Requests to access these datasets should be directed to Leah Estberg, leah.estberg@usda.gov.

## Author Contributions

JA wrote the first draft of the manuscript. LC and KM further revised the Introduction section. LE managed the database and downloaded the datasets used for summarization and assessment. HB performed the data assessment, created the tables and figures, and further revised the Results section. All authors contributed to the conception and design of the study, manuscript editing, and approved the submitted manuscript.

## Conflict of Interest

The authors declare that the research was conducted in the absence of any commercial or financial relationships that could be construed as a potential conflict of interest. The handling editor declared a past co-authorship with one of the authors JA.

## Publisher's Note

All claims expressed in this article are solely those of the authors and do not necessarily represent those of their affiliated organizations, or those of the publisher, the editors and the reviewers. Any product that may be evaluated in this article, or claim that may be made by its manufacturer, is not guaranteed or endorsed by the publisher.
